# Pharmacist-led medication reconciliation service for patients after discharge from tertiary hospitals to primary care in Singapore: a qualitative study

**DOI:** 10.1186/s12913-024-10830-6

**Published:** 2024-03-20

**Authors:** Konstadina Griva, Zi Yang Chua, Lester Yousheng Lai, Sandra Jialun Xu, Esther Siew Joo Bek, Eng Sing Lee

**Affiliations:** 1 Lee Kong Chian School of Medicine, Nanyang Technological University, Singapore, Singapore; 2 National Healthcare Group Pharmacy, Singapore, Singapore; 3 Clinical Research Unit, National Healthcare Group Polyclinics, 3 Fusionopolis Link Nexus@one-north (South Tower), #06-13, Singapore, 138543 Singapore

**Keywords:** Medication reconciliation service, Medication discrepancies, Medication review, Primary care, Transition of care, Discharge

## Abstract

**Background:**

Medication discrepancies commonly occur when patients are transferred between care settings. Despite the presence of medication reconciliation services (MRS), medication discrepancies are still prevalent, which has clinical costs and implications. This study aimed to explore the perspectives of various stakeholders on how the MRS can be optimized in Singapore.

**Methods:**

This is a descriptive qualitative study. Semi-structured interviews with 30 participants from the National Healthcare Group, including family physicians (*N* = 10), pharmacists (*N* = 10), patients recently discharged from restructured hospitals (*N* = 7) and their caregivers (*N* = 3) were conducted. All transcribed interviews were coded independently by three coders and inductive thematic analysis approach was used.

**Results:**

Five core themes were identified. (1) The MRS enhanced healthcare services in various aspects including efficiency and health literacy; (2) There were several challenges in delivering the MRS covering processes, technology and training; (3) Issues with suitable patient selection and follow-up; (4) Barriers to scaling up of MRS that involve various stakeholders, cross-sector integration and environmental restrictions; and finally (5) Role definition of the pharmacist to all the stakeholders.

**Conclusion:**

This study identified the role of MRS in enhancing healthcare services and explored the challenges encountered in the provision of MRS from family physicians, pharmacists, patients and their caregivers. These findings supported the need for a shift of MRS towards a more comprehensive medication review model. Future improvement work to the MRS can be conducted based on the findings.

**Supplementary Information:**

The online version contains supplementary material available at 10.1186/s12913-024-10830-6.

## Background

Medication is an important element of patient care and treatment of many diseases that requires constant review and optimization. Polypharmacy is defined as taking five or more medications daily [1]. While the global prevalence of polypharmacy varies from country to country, it has been found to be as high as 90% in the older adult population [2]. Medication management challenges often arise when patients are discharged from acute or tertiary institutions to the community and primary care settings, especially in the context of multimorbidity and polypharmacy [3]. Unintended changes to medications across prescribers may lead to discrepancies which can compromise patient safety and clinical outcomes [4].

According to the international consensus group, medication reconciliation service (MRS) is defined as “creating the most accurate list possible of all medication a patient is taking and comparing that list against the prescriber’s orders. In addition, the patients’ allergies and history of side effects from medications and medication aids are listed with the goal of providing correct medications to the patient at all transition points within the healthcare system” [5]. Similarly, the Ministry of Health in Singapore defines MRS as a structured and explicit process of creating the most accurate list possible of all medications a patient is taking, with the goal to ensure accurate and complete medication information transfer during transitions of care [6].

MRS has been provided in various settings, including home medicines reviews [7] as well as the more traditional service at tertiary settings. However, in Singapore, MRS is usually implemented at admission and discharge from tertiary healthcare settings and have shown to significantly reduce medication discrepancies [8]. The long-term sustainability of these benefits, however, may be limited as evidenced by the high readmission rate of older adult patients with medication discrepancies [8].

There is a need to complement MRS in the community and primary care. In Singapore’s primary care context, pharmacists are traditionally seen dispensing over the counter and they have generally been less involved in the clinical care of a patient. As such, MRS have not been widely adopted in primary care and patients referred to MRS also tended to be unfamiliar with the service. A recent review of the literature concluded that although pharmacists can resolve medication discrepancies with medication reconciliation at hospital discharge, patient outcome and care workload improvements were inconsistent [9]. Despite being able to resolve discrepancies, the MRS is also not widely adopted due to different barriers such as a lack of interprofessional communication [10, 11], patient’s resistance to having medicines reviewed, a lack of role clarity [10], and lack of standardized workflows [12]. Moreover, Weir et al. found that physicians were varied in their attitude towards MRS and they could be broadly divided into three groups, namely supportive, ambivalent and skeptical [13].

One study conducted at the National Healthcare Group Polyclinics (NHGP) in Singapore evaluated the value of pharmacist-led MRS in a primary healthcare setting for patients recently discharged from hospitals [14]. The study found that MRS reduced medication discrepancies in prescriptions by half and was subsequently implemented as part of standard care. Nevertheless, the efficacy of MRS to reduce medication discrepancies remains inconclusive [15]. Hence, there is a need for more studies on community-based MRS and its efficacy in complementing MRS conducted in the tertiary setting.

In addition to bridging MRS services across healthcare sectors, there is increasing recognition of the value of expanding the scope of these services to full medication review (MR) [16]. MR entails a structured, critical examination of a patient’s medicines with the objective of reaching an agreement with the patient about treatment, optimizing the impact of medicines, minimizing the number of medication-related problems, and reducing waste [17]. Both MRS and MR are components of medication management that will facilitate patients having the best outcome [6]. MRS is an important component of a MR. During a MR encounter, medication reconciliation is first performed before the structured review is conducted. While MRS would typically yield a list of medications and its associated doses that the patient is taking, a MR would involve additional reviews on medication suitability and optimization.

This study targeted on patients recently discharged from tertiary to the primary care setting. The focus was specifically on the existing MRS model in NHGP, and how it can be improved and expanded progressively towards a MR model. By gaining a better understanding of the stakeholders’ needs and concerns, we hope to develop a more comprehensive medication management model for the care and support of patients in primary care.

Hence, this study aimed to

1. evaluate the existing MRS model from the perspectives of healthcare providers, patients and caregivers.
2. describe the perspectives of the various stakeholders on how the MRS can be improved and further expanded.

## Methods

### Design

This study adopted a qualitative methodology involving semi-structured interviews with healthcare providers, patients, and caregivers. The study is nested in a larger study to develop a new medication reconciliation service (nMRS) in primary care. The qualitative study phase was mainly exploratory in nature and provided insights into the experience with existing MRS model (barriers and limitation, facilitators and value) and elicited feedback to guide the development of a better and improved MRS.

Ethics was approved by the National Healthcare Group (NHG) Domain Specific Review Board (DSRB) (reference number: 2018/01365). The COnsolidated criteria for REporting Qualitative research (COREQ) guidelines [18] were used to report the study.

### Setting and participants

The study was conducted from June to October 2019 at National Healthcare Group Polyclinics (NHGP). NHGP is a public primary healthcare institution that consists of seven polyclinics located in the northern and central parts of Singapore. Each polyclinic is a one-stop primary healthcare centre including pharmacy, laboratory and radiological services. All patients who were recently discharged from the hospital, aged more than 40 years old and referred to the polyclinics for follow-up were referred to the National Healthcare Group Pharmacy (NHGPh) for MRS in the polyclinics. These patients could be either new patients or existing patients of the polyclinics. They could be referred to the polyclinics for long-term follow-up of chronic diseases or short-term conditions such as resolving pneumonia. The MRS was only conducted once, for each discharge, during the transition from a tertiary hospital to primary care.

**Fig. 1:**
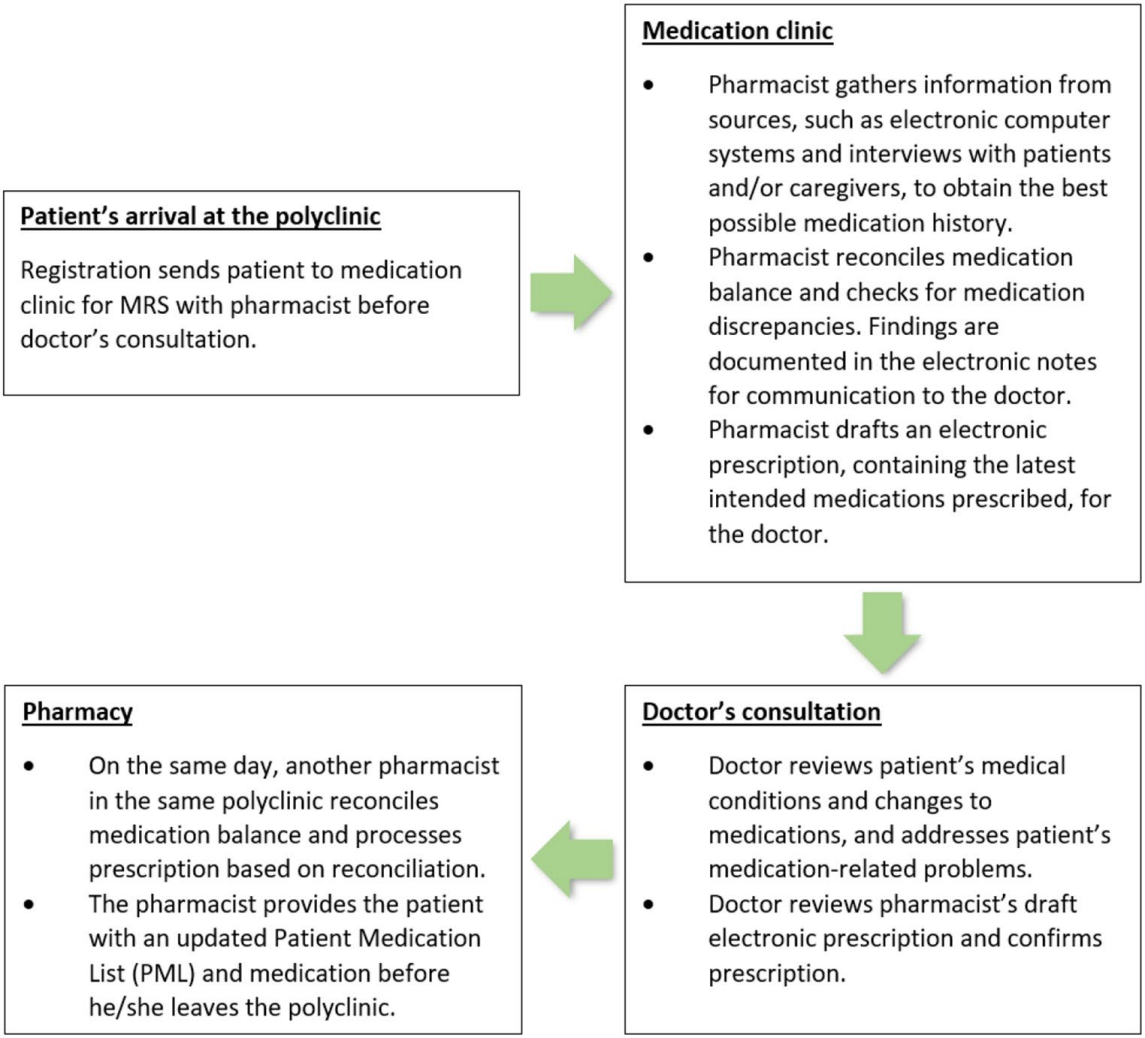
Patient’s journey through Medication Reconciliation Service (MRS) at National Healthcare Group Polyclinics (NHGP)

Figure 1 depicts the patient’s journey as they go through the MRS at NHGP. Briefly, appointment for MRS will be made for the patient upon discharge from tertiary care. All referred patients will attend the complimentary pharmacist-led MRS. During the MRS, which can last between 5 and 30 min, depending on the case complexity of the patient, a draft prescription will be prepared for the doctor. Further medication discrepancies were resolved after the consultation with the doctor, and the dispensing pharmacist will draft and provide the patient with a Patient Medication List (PML). The PML represents the most accurate list possible of prescribed and non-prescribed medications that a patient is taking at a particular point in time. The PML acts as a tool for communication with the next healthcare provider during transitions of care [6].

The study recruited healthcare providers that included pharmacists and doctors, and healthcare users that included patients and their caregivers into the study. Purposive sampling was adopted for the recruitment of healthcare providers to ensure a wide representation of experience and levels of clinical training. Convenience sampling was adopted for the recruitment of healthcare users. Eligibility criteria for the healthcare users were as follows: (a) aged 21 years and above, (b) able to converse in English or Mandarin, (c) recently discharged from tertiary care and attended at least one session of MRS at NHGP, and (d) willing and able to provide consent. A similar set of criteria was applied for the healthcare providers group with exception of (c). Healthcare users who were only able to converse in dialects or who were unable to give consent due to cognitive or psychiatric problems were excluded.

### Data collection

Eligibility for healthcare users was first ascertained by a pharmacist and potential participants were then approached by the research team members independent to patient care. Written consent was sought from all study participants and permission to use a recording device obtained prior to the commencement of the semi-structured interviews. Sociodemographic information including age, sex, and ethnicity, was collected during the interview. All healthcare users who agreed to participate in the study had the written consent and interviews conducted on the same day of MRS. Healthcare providers had the semi-structured interviews conducted at their preferred date and time. All interviews were conducted at the premises of NHGP.

Each interview lasted about 20 to 40 min and was conducted in the participants’ preferred language (English or Mandarin) by trained interviewers independent to the healthcare team. The two interviewers (Z.Y.C. and L.Y.L.) were trained qualitative researchers with undergraduate degrees in Psychology and were fluent in both English and Mandarin. Data collection and analyses were supervised by K.G.

Interviews were guided by topic guide developed with input from clinicians (E.S.L., E.S.J.B. and S.J.X.) and an experienced qualitative researcher (K.G.) (Supplementary Files 1 and 2). The topic guide was pilot tested and improved upon from the feedback received on relevance, clarity and acceptability of questions. The final topic guide for all participants were similar, comprising non-directive questions on the following topics: perceptions and experiences with current MRS services, concerns and challenges and recommendations for improvement. Interviews were audio recorded and transcribed verbatim. Non-English interviews were transcribed verbatim and translated into English. Transcripts were not returned to the participants.

### Analytical approach

An inductive thematic analysis approach [19] that uses the steps of familiarization, coding, theme development, reviewing themes, defining themes, and reporting was applied to identify barriers and facilitators. All transcribed interviews were coded independently by three coders (Z.Y.C., E.S.J.B. and L.Y.L.). Specialist software was not used. Initial codes and preliminary codebooks of emerging themes were iteratively refined after consensus were reached between coders. These preliminary codebooks were applied to all subsequent interviews. Two codebooks, one for healthcare providers and another for healthcare users, were developed and applied. Researcher reflexivity was supported by regular meetings with all team members in which themes (including illustrative quotes) and codebooks were reviewed and refined. All themes and codebooks were further reviewed and contrasted before a final master codebook was collaboratively developed, expanding and collapsing emerging themes. The final codebook was used to recode all transcripts. Coded quotes were organized by theme, subtheme, and participant type i.e., healthcare providers or users. Further recruitment of healthcare users and providers ceased when consensus was reached among research team members that data collected had reached thematic saturation.

## Results

A total of 20 healthcare providers were approached and all agreed to participate. A total of 20 eligible healthcare users were approached, of which 10 were referred and agreed to participate in the study. The study sample comprised *N* = 30 participants (Family physician, *N* = 10; Pharmacist, *N* = 10; Patient, *N* = 7 and Caregivers, *N* = 3). The clinical experience of pharmacists and family physicians ranged from three to 18 years and six to 35 years respectively. A total of 27 interviews were conducted in English and three in Mandarin. Table 1 depicts the demographics of the participants.

**Table 1:** Demographics of participants (*n* = 30)

		Healthcare providers (*n* = 20)		Health care users (*n* = 10)	
		*n*	*%*	*n*	*%*
Gender	Female	16	80	6	60
Age Group	20–30	3	15	0	0
	31–40	13	65	2	20
	41–50	3	15	0	0
	51–60	1	5	0	0
	> 60	0	0	8	80
Race	Chinese	17	85	8	80
	Malay	1	5	2	20
	Indian	2	10	0	0

**Fig. 2:**
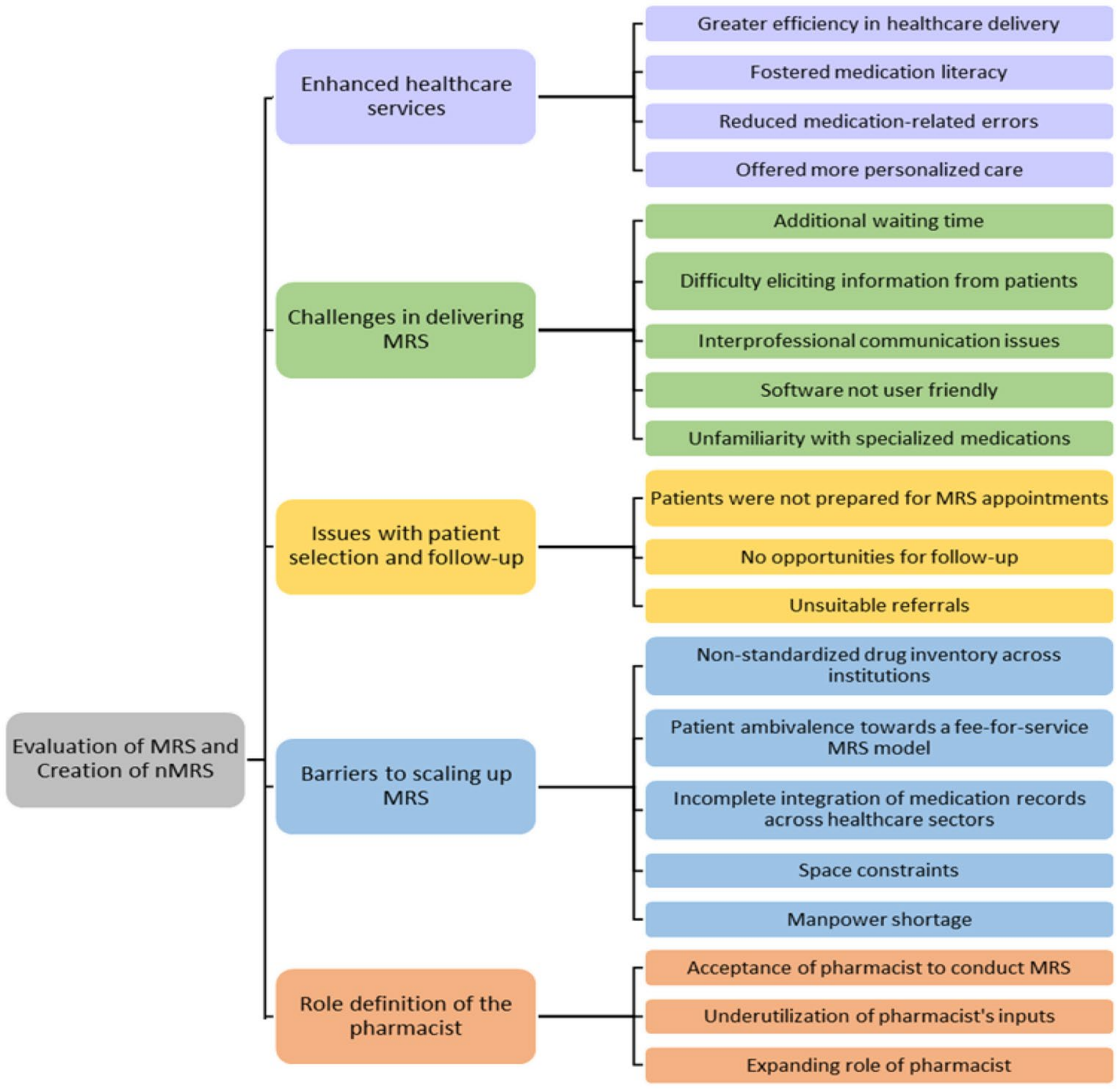
Thematic schema

The analysis revealed 20 themes that were grouped into five higher order themes: (1) Enhanced healthcare services, (2) Challenges in delivering MRS, (3) Issues with patient selection and follow-up, (4) Barriers to scaling up MRS, and (5) Role definition of the pharmacist. The higher order themes were generally comparable across healthcare providers and users and are presented in Table 2. Table 3 shows differing sub-themes unique to the healthcare provider group. The thematic schema is summarized in Fig. 2.

**Table 2:** Illustrative quotes from interviews with common themes

**(1) Enhanced healthcare services**	**Healthcare Providers**	**Healthcare Users**
Greater efficiency in healthcare delivery	*It’s very good because it helps us… the consultation time shorter because we don’t explain all these things and everything.* (MRS002, F)	*I guess all the screening thing is good for the doctor so that they can spend less time with us and spend more time looking after the patient.* (MRS022, M, Age 66)
Fostered medication literacy	*Another advantage is really sit down and explain to them the individual medicine and they will understand and they will actually take accordingly.* (MRS003, F, Age 39)	*Ah tell me everything about the medicine because I take WARFARIN… tell me how cannot eat this cannot eat this ah tell me everything about the medicine because I take WARFARIN ah then I cannot anyhow eat.* (MRS024, F, Age 69) *And the family member also can like if they also accompany him they can… they also be able to know what kind of medication the patient is taking.* (MRS030, F, Age 38)
Reduced medication-related errors	*Let’s say patient is new to the polyclinic, then probably the last seen hospital,… so they probably have to spend time to try to transcribe to our own system. So when transcribing, there may be chances of error… So at least when we draft for them, we have secured another additional layer…* (MRS019, M, Age 32)	*So, she actually did run through all the medication and updated me that it may not be those medicine that he is taking… that is having the side effect. It actually prompt me to go and alert the consultant later… and she also make a note in the system.* (MRS030, F, Age 38)
Offered more personalized care	*I think it’s good that the patient also gets some kind of, erm, recalibration of what they’re supposed to be taking and it’s good that it’s checked… Because sometimes, they’ll be yes yes yes you know, but actually they’re not taking the exact drug. So if somebody spends time to show them the medicine, then most likely they’d, you know.* (MRS014, F, Age 34)	*In depth like one-to-one session with the pharmacist, rather than you go over the counter which is very brief. I think it’s very personalized.* (MRS020, F, Age 34)
**(2) Challenges in delivering MRS**	**Healthcare Providers**	**Healthcare Users**
Additional waiting time	*They already waited so long in another consultation room, so they may refuse to come down for another consultation with the pharmacist, or they may think it’s more waiting time so that’s the only disadvantage.* (MRS002, F)	*If we can reduce the waiting time. Waiting time between seeing her and seeing the doctor.* (MRS030, F, Age 38)
**(3) Issues with patient selection and follow-up**	**Healthcare Providers**	**Healthcare Users**
Patients were not prepared for MRS appointments	*The patients are told to bring their medicines along so that we can do a physical medication reconciliation with them. But most of the time the patients either forget or they claim that they were not told.* (MRS005, F, Age 38) *Because a lot of patients they come here, actually they don’t know what the clinic for. They come here “eh why I come to see the pharmacist?”.* (MRS001, F, Age 31)	*I actually thought that it was… a blood test because usually we don’t have an appointment before consultation. And whatever appointment taken before consultation should either be blood test or X-ray or whatever. So, I thought it was a blood test… it is all housed in the same polyclinic. It is just different levels.* (MRS030, F, Age 38)
No opportunities for follow-up	*Because… ok we can only do that if the patient comes to collect medicine. So, if the patient do not come to collect medicine then if there’s changes in medicine then we cannot reinforce.* (MRS003, F, Age 39) *Because currently it’s a once off we see the patient one time and then most probably we won’t see the patient again unless they are readmitted.* (MRS005, F, Age 38)	*Follow up, probably if the system is able to detect a change in the patient medication, after doctor may have eh included, add in new ones or stop existing ones like that so if there is a change probably the system can prompt the pharmacist to… when there is a change in medication.* (MRS030, F, Age 38)
**(4) Barriers to scaling up MRS**	**Healthcare Providers**	**Healthcare Users**
Non-standardized drug inventory across institutions	*But of course when we do get a lot of brand changes, different institutions carry different brands, that’s the hard part as well… So like you have to start guessing.* (MRS011, M, Age 28)	*Yeah! tally or not or generic, cause X hospital and this Y hospital different company…* (MRS028, M, Age 67)
Patient ambivalence towards a fee-for-service MRS model	*Mmm, tsk, I tell you… the truth is if the patient need to pay for my service right, they probably won’t want my service…* (MRS004, F, Age 32) *now I don’t think they are charging, but I think next time if they were to impose any charges, then it’s probably convincing the patient to see them.* (MRS018, F, Age 32)	*I don’t think we should be made to pay, cos this is a service.* (MRS029, M, Age 72) *If they were to charge, I mean I don’t mind… if it’s a few dollars I am still ok. Affordable…, some of them may not be affordable…, not for me I don’t mind.* (MRS025, F, Age 65)
**(5) Role definition of the pharmacist**	**Healthcare Providers**	**Healthcare Users**
Acceptance of pharmacist to conduct MRS	*Oh, the reason is because I think if the med recon is to go through medication, erm, and then, go through doses and to have the NEHR access to double check, it falls within the scope of what the pharmacist does…* (MRS013, M, Age 33)	*Yup yup definitely that will help yup I think that is her specialization… so she will know which pharmaceutical is famous for doing medicine that kind of thing so I think… should take her recommendation… that is her area of specialty yes… I would think she can do that.* (MRS030, F, Age 38)

We identified sub-themes within four of the main themes that were unique to healthcare providers and presented them in Table 3 below.

**Table 3:** Illustrative quotes from interviews with sub-themes unique to healthcare providers

**(1) Challenges in delivering MRS**	**Healthcare Providers**
Difficulty eliciting information from patients	*Then the other one is certain patient they might refuse to reveal more due to certain psychosocial issue in which umm… we can try our best to elicit the correct information.* (MRS010, F, Age 33) *Umm…if patient come alone, and then they themselves don’t know what they taking, or someone pack for them. Then they have no idea at all.* (MRS008, F, Age 43)
Interprofessional communication issues	*Like, a doctor might be with a patient and the same time, the pharmacist wants to clarify something. So, if the pharmacist wants to clarify this and wants to communicate directly with the doctor, the doctor may be a bit busy to do that. And sometimes there can be a bit of tension.* (MRS013, M, Age 33) *because we draft the ERX [prescription] on what the patient is supposed to be taking but then we will put in the remarks patient is taking like one tablet in the morning instead of two tablets in the morning right, then some doctors they do not look at the remarks then they just press approve.* (MRS003, F, Age 39)
Software not user friendly	*The IT system can be… improved and reduce the time to generate a PML [patient medication list] or… can help to smoothen the workflow then I think will be better. I think IT currently is the main thing and the PML actually when we need to generate is quite manual and need to key in the… indication one by one.* (MRS010, F, Age 33)
Unfamiliarity with specialized medications	*If I’m not familiar with the medicine, I feel like that’s the barrier. Because I’m very familiar with whatever we have here in the pharmacy, but some medication maybe is not my specialty, I might not be familiar.… I feel I cannot advise on that.* (MRS016, F, Age 29)
**(2) Issues with patient selection and follow-up**	**Healthcare Providers**
Unsuitable referrals	*I think that’s where the frustration come in you know, like I already so busy outside [pharmacy area], you come in for the med clinic then after that the patient doesn’t even have chronic medicine then waste my time*. (MRS003, F, Age 39)
**(3) Barriers to scaling up MRS**	**Healthcare Providers**
Incomplete integration of medication records across healthcare sectors	*Especially from the hospital, sometimes we can’t see the hospital medicine list. Because… private prescription and other things we can’t… it’s not connected.* (MRS015, F, Age 38)
Space constraints	*Cos we got no [extra] rooms, so even if we have pharmacist we cannot do [medication reconciliation service].* (MRS003, F, Age 39)
Manpower shortage	*But for the workflow, sometimes when down on manpower for the pharmacist we still need to allocate one pharmacist to do this service, this one is part of the service…* (MRS010, F, Age 33)
**(4) Role definition of the pharmacist**	**Healthcare Providers**
Underutilization of pharmacist’s inputs	*And… perhaps if the clinicians…, don’t know that the drafted list is meant to be worked upon as the current thing and they just decide to prescribe everything from the beginning and redo, then… it will actually…, have wasted the effort of the pharmacist as they go through the recon list…* (MRS013, M, Age 33) *sometimes the doctors like don’t see our, don’t see our list or don’t see our notes. So… I feel that our handover is not very good so there are times where I think the physician don’t see our notes.* (MRS004, F, Age 32)
Expanding role of pharmacist	*So right now is merely just… checking adherence and… any other medication-related problems but we can’t really suggest optimization because we don’t have the labs with us.* (MRS005, F, Age 38) *Or if there’s a polypharmacy for the elderly can we actually cut down the medicine or optimize the medicine usage which is actually not part of the med clinic?* (MRS003, F, Age 39) *… because number 1… they (the pharmacists) are able to take the responsibility of it being right or wrong. It’s… similar to doctors taking the responsibility of diagnosing something.* (MRS012, F)

### Enhanced healthcare services

The existing MRS was generally well received by both healthcare providers and users. The MRS reduced the burden of care from the physicians by leveraging the inputs of the pharmacists. Both groups of providers perceived an increase in efficiency of healthcare delivery through the MRS by optimizing the physician’s consultation time and using the MRS as a platform to discuss medication-related problems. There were also less re-work for the pharmacists during dispensing.

Healthcare users appreciated the service as they could raise concerns with better knowledge of their own medications, helping them to understand why they were prescribed certain medications and the importance of adherence. Pharmacists utilized the platform to check on patients’ medication adherence, discrepancies in the medication list and foster health literacy.

The MRS was also perceived to improve medication safety by reducing medication-related errors through the provision of an additional layer of checks when the pharmacists conducted the MRS. Finally, healthcare users also could be alerted to their own incorrect medication-taking behaviour. The personalized service and the involvement of caregivers in medication management were welcomed.

### Challenges in delivering MRS

There were several factors identified that impeded the delivery of the MRS, and these were predominantly experienced by the healthcare providers. These factors can broadly be categorized as patient-related and non-patient-related issues. Patient-related issues included an additional waiting time incurred by healthcare users and difficulty in eliciting information useful to the MRS session.

The MRS added to the overall amount of time a patient was required to spend in the polyclinic. This was a result of having to move from room to room for each different service and the time required to wait their turn for each service provider. The additional waiting time was the only challenge that was cited by both healthcare providers and users.

During the MRS, some patients might not be entirely forthcoming when providing pharmacists with information or were simply forgetful. Other patients may not come along with a caregiver familiar with their medications or may not have brought along their medications for a meaningful MRS to be carried out.

Non-patient-related factors included interprofessional communication, issues with the technology used and unfamiliarity with specialized medications. The internet separation from workstations that was enforced since 2018 hampered interprofessional communication as healthcare providers lost the ability to communicate with each other over electronic messaging systems and had to communicate via phone calls instead. These phone calls were perceived by physicians to be interruptive to the consultation process and could potentially lead to tension between physicians and pharmacists. Pharmacists also felt that physicians did not fully utilize the prescriptions drafted for them and did not read or take action from their notes.

The inability of healthcare providers to connect to the internet on clinic computers and user-unfriendly software systems contributed to further challenges faced by the pharmacists. In order to carry out the MRS, pharmacists often had to use multiple electronic systems which were unlinked. Some pharmacists also reported unfamiliarity with medications prescribed by specialist physicians in the hospitals when conducting the MRS.

### Issues with patient selection and follow-up

Patients and their caregivers were not prepared for MRS appointments and lack of opportunities for follow-up were common sub-themes raised. A high default rate was reported for MRS and this was perceived to be due to missed or inadequate appointment notification reminders and users’ lack of awareness on the purpose of the MRS appointment.

Pharmacists reported the lack of patient tracking after MRS being a wasted opportunity to monitor outcomes. Pharmacists often did not get to know the outcome of their recommendations and were not given the opportunity to follow-up with patients to check if their medication issues picked up during the MRS had been resolved. Healthcare users also expressed interest in having follow-up MRS consultations when there were changes to their medication plan.

Pharmacists reported high frequency of unsuitable patients being referred for MRS. These included patients with no medication changes, had a good grasp of the medication regimens and patients who did not have polypharmacy. Likewise, patients with no medication-related problems did not see the need to attend MRS.

### Barriers to scaling up MRS

Drug inventories across different institutions were not standardized and affected prescription and reconciliation of medications. This issue was also compounded by frequent brand changes in medications stocked in the different institutions.

The MRS is currently provided as a complimentary service and would not be sustainable in the long-term. However, many healthcare providers felt that users would be unlikely to support MRS if it were a paid service. Most healthcare users valued the cost for MRS at approximately SGD$10 but reflected reluctance towards a fee-for-service model.

Healthcare providers also expressed the incomplete integration of medication records across different health sectors resulting in difficulties accessing patients’ medication history. Therefore, scaling the MRS to include patients discharged from a wider spectrum of institutions was deemed to be problematic.

Additionally, healthcare providers also expressed concerns over logistical requirements for scaling up of MRS in NHGP. The conduct of MRS requires a quiet environment and there is currently a shortage of suitable rooms in the polyclinics to be dedicated for MRS use. Likewise, the shortage of manpower was a common concern. To provide MRS, manpower has to be taken away from the already busy frontline at the pharmacy department.

### Role definition of the pharmacist

There was agreement among all participants that pharmacists were the most suitable healthcare provider to conduct the MRS. Other suitable candidates suggested were pharmacy technicians, nurses, clinical managers and clinical coordinators to conduct MRS with adequate training.

Responses were mixed when discussing how the role of the pharmacist could be expanded. Healthcare providers, in general, were confident that the role of the pharmacist could be expanded to include optimizing medication usage and ordering laboratory tests, like a medication review model [20]. Deprescribing, the process of tapering, stopping, discontinuing and withdrawing drugs, with the goal of managing polypharmacy and improving outcomes, was also mentioned as a possible role of a pharmacist [21]. However, it was pointed out that a medication review model will require greater responsibility from the pharmacists and concerns were raised over the lack of medicolegal protection for the pharmacists. Further concerns were also raised across healthcare providers, over the liability of the prescription, which was drafted by the pharmacist but authorized by the physician.

## Discussion

This study sought to elucidate the perspectives of healthcare providers, patients and their caregivers on the existing Medication Reconciliation Service (MRS) model in National Healthcare Group Polyclinics (NHGP) and how the service could be improved. Five themes emerged that captured how the MRS enhanced current healthcare services, perceptions on the challenges encountered, patient selection and follow-up, barriers to scaling up the service, and differing perspectives on the role of pharmacists.

Consistent with a study by Siaw et al. [22], the pharmacist-led MRS was perceived by healthcare providers and users to improve healthcare service delivery with a reduction in physician burden. Healthcare users in our study appreciated the personalized, caregiver-involved MRS as a platform to raise medication-related concerns and perceived a better understanding of their medication. The sentiments of improving medication knowledge in patients and caregivers, which translates to improved health literacy were echoed by healthcare providers. Enhancing patient’s health literacy has been identified as a crucial factor for optimizing medication reconciliation [23]. Routine reviews of medication can improve health literacy [24]. Pharmacist-led MRS has been shown to be effective in reducing medication discrepancies [25] thus reducing medication errors and improving medication safety. This perspective was common for both healthcare providers and users. The involvement of pharmacists in hospital discharge transitions also demonstrated positive effects in decreasing inpatient readmissions and emergency department visits in high-risk patients [26].

Healthcare providers described several challenges including difficulty eliciting information from patients and their caregivers, software and hardware issues hampering interprofessional communication and their unfamiliarity with hospital medications that were seldom used in the primary care setting. Patient selection criteria from the hospital for primary care MRS was not very clear and healthcare users were not adequately prepared or often forgot about the instructions given to them to attend the MRS with their main caregiver or to bring all their medications for the session.

When exploring on how the current MRS could be improved or scaled up further, healthcare providers highlighted barriers such as the use of different brands of similar drugs across institutions, incomplete integration of information across institutions especially with private healthcare institutions, space and manpower constraints in providing MRS and concerns about the sustainability of the complimentary service provision. A study done among community pharmacist in the United States also highlighted similar barriers in performing medication reconciliation including pharmacy resources and the lack of helpful information such as hospital discharge lists [27].

There was unanimous agreement that pharmacists were the most suitable professionals for MRS. Pharmacists felt that the current MRS did not fully utilize their expertise and aspired for their roles to be expanded. There was consensus that MRS allowed for better efficiency in healthcare delivery, fostered better medication literacy, provided personalized care to healthcare users and reduced medication-related errors.

Challenges encountered in eliciting accurate information from patients during MRS was a common issue encountered. Older patients usually have one main caregiver who helps in the administration of medications. However, this caregiver may not accompany the patient on the day of the MRS appointment. Without the main caregiver present, the pharmacist conducting the MRS was not able to determine the patient’s medication adherence. Some patients also did not bring the medications dispensed to them at discharge to the MRS. Without the physical medication, it was difficult for pharmacists to accurately determine a patient’s medication taking behaviour. While the pharmacist aims to establish a most accurate list of medications that the patient is taking during the medication reconciliation process, a challenge that persists in ensuring this accuracy is the patient’s own driven changes to the medication regimen [28]. The pharmacist may attempt to show samples of the medication but the non-standardized drug inventory across institutions makes this solution less useful. The patient may have been prescribed a medication that is not stocked in the primary care setting thus there may not be a sample available to show the patient. Institutions stocking the same medications may not always carry the same brands thus affecting the uniformity of the appearance of the medication. This results in the patient or caregiver not being able to recognize the medication that the pharmacist is showing.

Ineffective targeting and tracking of patients resulted in unsuitable patients being referred for MRS and conversely, patients who could benefit from the MRS were not. From the perspectives of the healthcare providers, the geriatric population, patients with polypharmacy and chronic conditions should be the commonly reported target population of MRS. Other healthcare users may not reap the full benefits of MRS. These included patients with no changes to their medications, no chronic diseases, good knowledge about their medications and few medications to manage. They would divert limited resources from other healthcare users who would more likely benefit from the service.

Some healthcare users also reported not being aware as to the purpose of the MRS, resulting in confusion as to why an additional service station was added to their visit itinerary Others expressed surprise as to the additional appointment to another healthcare provider and lamented the extra time incurred. Healthcare providers expressed concerns over how appointments were relayed to patients. Patients were informed of their MRS appointments via text messages. However, some patients did not update their contact information leading to them not being notified and others may not understand English language text messages. In order for patients to benefit maximally from the MRS, a better system which can prepare patients before the day of their MRS appointment can circumvent these issues and remind them to attend the MRS with their main caregiver with all their home medications.

The National Electronic Health Records (NEHR) is Singapore’s attempt since 2009 to integrate all electronic healthcare records throughout the nation with a centralized platform [29]. Healthcare providers found that the incomplete integration of medication records across healthcare sectors to be a challenge. The NEHR contains only records of healthcare information from public institutions. As such, a patient’s healthcare information is incomplete if he visits private institutions. This poses significant challenges to the pharmacist who is trying to obtain the best possible medication history. Ironically, it is this exact challenge that is the very reason why MRS is pertinent, especially in the transition of care from tertiary to primary care setting.

Health information system integration is a daunting task, and numerous information-system related challenges were reported in a study of health systems reforms in Singapore, including the difficulties integrating the NEHR across different healthcare clusters and providers [30]. Ten years later, the persistence of health systems-related problems as per our study findings reiterate the complexity of healthcare integration. A major challenge to healthcare integration was the difference in power, legitimacy and urgency each healthcare stakeholder has, resulting in a different uptake rate of NEHR across healthcare institutions [30]. Health system problems raised in our study stems from the difference in motives, priorities and philosophies of healthcare stakeholders [31]. This issue of adopting electronic health records is not unique to Singapore. In the United States, about 1 in 4 hospitals have not adopted electronic health records and some barriers to the adoption included initial costs of adoption, technical concerns, technical support and resistance to change [32] We understand that NEHR has made further progress since the completion of the study to include prescription and laboratory test results from private hospitals and private general practitioners.

Like a study by Low et al. [33], healthcare users in our study were comfortable with sharing their health needs with the pharmacists. However, there was a divergence in opinions when asked about expanding the role of the pharmacist for the MRS. Despite the acceptance of a pharmacist-led MRS, some healthcare providers felt that there was an underutilization of pharmacist’s expertise. Most physicians used the pharmacist’s prescription draft as a template. However, there were occasions where prescriptions drafted by pharmacists were entirely different from the prescriptions drafted by the physicians with no further feedback. As such, the pharmacists concerned were uncertain if their prescriptions were drastically amended or totally ignored. During the in-depth interviews, some physicians also reported that they had forgotten all about the existence of MRS and therefore missed out on the pharmacists’ inputs.

Despite the general acceptance of the pharmacist-led MRS by healthcare users, there were concerns on the scope of duties the pharmacist could perform. Healthcare users viewed pharmacists as an advisor on the indications of their medications and their usage. However, they did not feel comfortable for pharmacists to have the rights to prescribe or change their medications without the authorization of a physician and expressed that medication changes should only be made under the authority of the physician. On the other hand, healthcare providers perceived deprescribing and medication optimization to be within the scope of work of the pharmacists. This finding was in contrast with a scoping review where the public generally supported pharmacist prescribing in situations such as chronic conditions and minor ailments [34]. This may be because most of the public in Singapore were unaware that pharmacists are trained to attend to medication queries and optimize drug therapy [32]. Therefore, it is necessary to educate patients and caregivers on the knowledge, skills and professional abilities of pharmacists in Singapore to improve the acceptability among healthcare users.

A major strength of our study was the inclusion of all stakeholders (family physicians, pharmacists, patients and caregivers) in the evaluation of the MRS. Conversely, there were several limitations to our study. A convenience sample of healthcare users (with only 10 out of 20 approached) participated in the study. Patients who were not fluent in English or Mandarin were excluded from the study due to the language competencies of the research team. In addition, the study did not include healthcare users who chose not to receive MRS, providers who decided not to participate in the initiative and policymakers who initiated the pharmacist-led MRS program. The other limitation to this study is the applicability of these themes to our current healthcare landscape as the interviews were conducted in 2019. Since the interviews in 2019, there have been minor tweaks to the MRS but we feel that the findings of this study would still be very relevant to the various stakeholders including policymakers of MRS and the wider system overseas with similar pharmacist-led services.

## Conclusions

In conclusion, the findings supported the enhancement of healthcare delivery brought by the pharmacist-led MRS. However, issues in the engagement of patients and systemic barriers to MRS delivery were uncovered from the study. Cross-institutional efforts and engagement of stakeholders are vital for scaling operationalization of MRS. Acceptability of expanding the role of the pharmacist is an essential component to factor in during the roll-out of the improvement of current MRS and the birth of future medical review service. The findings of the study is not novel but are confirmatory to what others have found in other healthcare systems. We conclude that while the issues identified for services to reduce medication errors are probably similar in different healthcare systems, the solutions and measures to improve them are nuanced to each of the different systems and would require a whole of system approach to tackle them. Future work would involve sharing of the successes and failures of such interventions.

### Electronic supplementary material

Below is the link to the electronic supplementary material.

Supplementary Material 1

Supplementary Material 2

## Data Availability

All data generated or analysed during this study are included in this published article.

## Abbreviations

COREQ: Consolidated Criteria for Reporting Qualitative Research
DSRB: Domain Specific Review Board
MR: Medication Review
MRS: Medication Reconciliation Service
NEHR: National Electronic Health Records
NHG: National Healthcare Group
NHGP: National Healthcare Group Polyclinics
NHGPh: National Healthcare Group Pharmacy
nMRS: New Medication Reconciliation Service
PML: Patient’s Medication List
