# Supplementary material for: Pharmacist-led medication reconciliation service for patients after discharge from tertiary hospitals to primary care in Singapore: a qualitative study
Source: BMC Health Serv Res. 2024 Mar 20;24:357. doi: 10.1186/s12913-024-10830-6 (PMC10956343; doi:10.1186/s12913-024-10830-6)
Supplement: Supplementary file 1 — Supplementary Material 1 [file 12913_2024_10830_MOESM1_ESM.docx]

**Developing the ideal collaborative Medication Management Model for patients during transition to primary care – a pilot study**

**Topic Guide (Patient/Caregiver)**

| **The current MRS service** | You went through the medication reconciliation service before you saw the doctor.  Can you tell me about your experience with the MRS?  *What did you like about it? What did you not like as much and can be improved on?*   - Environmental factors (e.g. location, amount of time taken) - The process (e.g. interview process, language used) - Workflow (e.g. before seeing the doctor) - Others |
| --- | --- |
| **What would their ideal MRS look like?** | Have you gone through something similar at other healthcare institutions such as in the hospital? *(Compare experiences between the different services)*  Describe what an ideal service is in your own view. What should the service do?  Is the MRS done at an appropriate timing in the patient’s journey through the clinic? (It is currently being done by the pharmacist before a doctor’s consult.) |
| **Whose role is it to do medication reconciliation?** | There are numerous healthcare professionals that are involved in the care of a patient. This includes the doctors who prescribe medications, pharmacists who dispense the medications as well as nurses who provide care for the patients.  In your opinion, which healthcare professional is most suited to conduct a MRS? |
| **What are the patient’s views on the medication reconciliation service?** | Which patients would benefit most from a MRS?   - Age - Gender - Ethnicity - Education Level - Multiple prescribers - Multiple healthcare institution visits   How important do you think this service is for you as a patient?  How much would you pay for this service? |
