# Supplementary material for: Pharmacist-led medication reconciliation service for patients after discharge from tertiary hospitals to primary care in Singapore: a qualitative study
Source: BMC Health Serv Res. 2024 Mar 20;24:357. doi: 10.1186/s12913-024-10830-6 (PMC10956343; doi:10.1186/s12913-024-10830-6)
Supplement: Supplementary file 2 — Supplementary Material 2 [file 12913_2024_10830_MOESM2_ESM.docx]

**Developing the ideal collaborative Medication Management Model for patients during transition to primary care – a pilot study**

**Topic Guide (Healthcare Provider)**

| **The current MRS service** | What do you think of the current MRS?  What are the advantages or disadvantages of the medication reconciliation service?  What do you like about it? What do you not like as much and can be improved on?   - Environmental factors (e.g. location, amount of time taken) - The process (e.g. interview process, language used) - Workflow (e.g. before seeing the doctor) - Others   What resources do you need to do the MRS? What is currently lacking and what other resources would you need? Are they available?   - Manpower - Support - Time - System |
| --- | --- |
| **What would their ideal MRS look like?** | Describe what an ideal service is in your own view  Is the MRS done at an appropriate timing in the patient’s journey through the clinic? (It is currently being done by the pharmacist before a doctor’s consult.) |
| **Whose role is it to do medication reconciliation?** | There are numerous healthcare professionals that are involved in the care of a patient. This includes the doctors who prescribe medications, pharmacists who dispense the medications as well as nurses who provide care for the patients.  In your opinion, which healthcare professional is most suited to conduct a MRS? |
| **What are the HCP’s views on the medication reconciliation service?** | Which patients would benefit most from a MRS?   - Age - Gender - Ethnicity - Education Level - Multiple prescribers - Multiple healthcare institution visits   How do you or your colleagues feel about the MRS?   - Usefulness/importance - Resistance faced by other healthcare professionals - Professional roles and boundaries - Confidence in conducting the MRS - Intangibles e.g. recognition by colleagues/patients, time needed - Feedback (positive or negative) |
